# Targeting Natural Killer Cells for Tumor Immunotherapy

**DOI:** 10.3389/fimmu.2020.00060

**Published:** 2020-02-19

**Authors:** Cai Zhang, Yuan Hu, Chongdeng Shi

**Affiliations:** Institute of Immunopharmaceutical Sciences, Key Laboratory of Chemical Biology (Ministry of Education), School of Pharmaceutical Sciences, Shandong University, Jinan, China

**Keywords:** natural killer cells, tumor microenvironment, cytokine, CAR-NK, checkpoint blockade, tumor immunotherapy

## Abstract

Natural killer (NK) cells are important innate cytotoxic lymphocytes with a rapid and efficient capacity to recognize and kill tumor cells. In recent years, adoptive transfer of autologous- or allogeneic-activated NK cells has become a promising cellular therapy for cancer. However, the therapeutic efficiency is encouraging in hematopoietic malignancies, but disappointing in solid tumors, for which the use of NK-cell-based therapies presents considerable challenges. It is difficult for NK cells to traffic to, and infiltrate into, tumor sites. NK cell function, phenotype, activation, and persistence are impaired by the tumor microenvironment, even leading to NK cell dysfunction or exhaustion. Many strategies focusing on improving NK cells' durable persistence, activation, and cytolytic activity, including activation with cytokines or analogs, have been attempted. Modifying them with chimeric antigen receptors further increases the targeting specificity of NK cells. Checkpoint blockades can relieve the exhausted state of NK cells. In this review, we discuss how the cytolytic and effector functions of NK cells are affected by the tumor microenvironment and summarize the various immunotherapeutic strategies based on NK cells. In particular, we discuss recent advances in overcoming the suppressive effect of the tumor microenvironment with the aim of enhancing the clinical outcome in solid tumors treated with NK-cell-based immunotherapy.

## Introduction

The important innate cytotoxic lymphocytes, natural killer (NK) cells, show rapid, and efficient cytolytic activity to recognize and kill both virally infected and transformed cells. They exert effector functions relying on germline-encoded receptors for their activation, without the need for prior exposure to the antigen. NK cells possess an extensive repertoire of receptors (activating and inhibitory) that recognize altered protein expression on target cells, thereby controlling the cytolytic function. Uniquely, these receptors can distinguish normal from transformed cells via “missing-self” or “induced self” recognition models (1, 2). The balance between activating and inhibitory receptors tightly governs the inhibition or activation of NK cells, and inhibitory signals from inhibitory receptors usually predominate over activation signals for the maintenance of homeostasis (3, 4). NK-cell-activating receptors recognize stress-induced molecules on target cells to prime NK cell activation. The downregulation of inhibitory ligands and the expression of ligands that activate receptors on tumor cells trigger the activation of NK cells to kill these abnormal cells. Both human and mouse NK cells express a CD16-activating receptor, which mediates antibody-dependent cellular cytotoxicity (ADCC) upon binding to the Fc portion of antibodies. To stimulate responses to target cells, NK-cell-activating receptors, such as NK group 2D (NKG2D), CD226, and the natural cytotoxicity receptors NKp30, NKp44, and NKp46, are considered the most relevant receptors. Once activated, NK cells lyse tumor cells through the release of the cytotoxic molecules perforin and granzyme, upregulating the expression of the Fas ligand and tumor necrosis factor-related apoptosis inducing ligand, and the secretion of cytokines such as interferon gamma (IFN-γ) and TNF alpha (TNF-α).

In addition to their direct cytotoxic potential against target cells, NK cells also exhibit immunoregulatory functions. NK cells contribute to the homeostasis of the immune system and initiate or promote the activation and effector functions of other innate and adaptive immune cells via secretion of cytokines and chemokines, or through direct cell–cell contact (5, 6). NK cells can shape adaptive immune responses through cross-talk with other immune cells such as T cells, B cells, and dendritic cells (DCs). NK cells promote Th1 polarization by producing IFN-γ. They also indirectly enhance the immune responses of adaptive T cells by promoting DC maturation (6, 7). Emerging evidence suggests that NK cells enhance CD8^+^ T cells function and/or ameliorate their exhaustion (8–10). NK cell function is proven to be critical for the antitumor or antiviral effects of CD8^+^ T cells, even with checkpoint blockade therapy (11).

Recently, it was found that NK cells have features of adaptive immune cells, particularly the memory-like responses (12–18). A long-lived murine NK cell population had been reported to be able to self-renew and survive in a lymphopenic environment for more than 6 months (19). To date, three types of long-lived memory NK cells have been identified: hepatic-liver-resident NK cells, cytomegalovirus (CMV)-specific NK cells, and cytokine-induced memory-like NK cells (20–22). They usually express high levels of NKG2C and have the capacity for long-term *in vivo* proliferation and persistence. Upon restimulation with antigens or cytokines, memory-like NK cells undergo clonal-like expansion followed by longevity, self-renewal, and recall responses (13, 23, 24). Recently, the transcription factor interferon regulatory factor 8 has been found to orchestrate the adaptive NK cell response against CMV infection (25). A recent study showed that naive NK cells could be induced to functionally convert into tumor-induced memory-like NK cells by priming using acute myeloid leukemia or pediatric acute B-cell leukemia specimens (14). These tumor-induced memory-like NK cells exhibit certain similarities to cytokine-induced memory-like NK cells and CMV-specific NK cells; however, more importantly, they show significant differences, such as higher tumor-specific cytotoxicity and increased synthesis of perforins, but not IFN-γ secretion. These NK cell adaptive features are promising for the future use of immunotherapy to treat cancers and infective diseases.

NK cells' critical role in immunosurveillance and their powerful antitumor efficacy have prompted their use in many clinical trials to control tumor growth via their effector capacity. However, although the results have been encouraging in hematological malignancies, there has been less success for solid tumors. Indeed, solid tumors present considerable challenges to the application of NK-cell-based therapies. For example, it is difficult for NK cells to traffic and infiltrate into the tumor sites. NK cell function, activation, and phenotype are impaired by the tumor microenvironment, even rendering NK cells dysfunctional or exhausted. Thus, strategies to improve the cytolytic activity, durable persistence, and activation of NK cells have been developed. In the present review, we discuss how the cytolytic and effector functions of NK cells are affected by the tumor microenvironment. We also summarize the various immunotherapeutic strategies based on NK cells, especially the recent attempts to improve NK-cell-based immunotherapy clinical outcomes against solid tumors by overcoming the suppressive effect of the tumor microenvironment.

## Effect of the Tumor Microenvironment on NK Cells' Cytolytic Function

NK-cell-based immunotherapies, particularly the adoptive transfer of autologous or allogeneic NK cells, or gene-modified NK cells, have been used widely in clinical trials and have shown great promise for different hematological malignancies (26, 27). However, for patients with solid tumors, the outcomes of adoptive NK cell infusions have been disappointing. There are considerable challenges for NK cell therapy to treat patients with solid tumors. One of the major challenges is the difficulty of NK cells to traffic to the tumor location and infiltrate into the tumor. This poor ability of NK cells to infiltrate into solid tumors limits the clinical outcome of adoptive NK cell infusion. Enhanced infiltration of NK cells into tumor lesions has been associated with good prognosis for patients with diverse types of solid cancer (28, 29).

Another major challenge comes from the tumor microenvironment, which impairs the phenotype, activation, persistence, and function of NK cells. Accumulating data have shown that tumor-infiltrating NK cells exhibit poor cytotoxic capacity, accompanied by downregulation of activating receptors and upregulation of inhibitory receptors, compared with NK cells in non-tumor tissues (4, 30, 31). The tumor microenvironment is a complex network comprising regulatory T cells (Tregs), tumor-associated macrophages (TAMs), regulatory γδT cells, myeloid-derived suppressor cells (MDSCs), soluble factors, the extracellular matrix, and suppressive molecules expressed on tumor cells (32–35). NK cell proliferation and antitumor activity are suppressed by tumor cell secretion of various immunosuppressive factors, including prostaglandin E2, indoleamine 2,3-dioxygenase (IDO), interleukin 10 (IL-10), transforming growth factor-β (TGF-β), and vascular endothelial growth factor. The growth of many types of solid tumors promotes the expansion of immunosuppressive cells, including Tregs, MDSCs, and TAMs. Tumor cells also secrete chemokines, such as C–X–C motif chemokine ligand 8 or C–C motif chemokine ligand 2, to promote Tregs, TAM, or MDSCs accumulation at the tumor sites. By producing TGF-β and IL-10, or via direct cell-to-cell interaction, these immunosuppressive cells inhibit intratumoral NK cell cytotoxicity (36–38). Tregs directly inhibit NK cell cytolytic functions via the production of TGF-β and also reduce the expression of activating receptors NKG2D and natural cytotoxicity triggering receptor 3 (NKp30) through membrane bound TGF-β (39, 40). MDSCs suppress NK cell cytotoxicity and cytokine secretion via membrane-bound TGF-β on MDSCs in a cell-contact-dependent manner or dependent on NKp30 on NK cells (39, 41). Several proinflammatory cytokines have also been reported to contribute to MDSC-mediated NK cell inhibition, resulting in reduced NK cytotoxicity, decreased expression of activating receptor NKGD or NKR, and limited release of IFN-γ (42). These soluble factors include TGF-β, IDO, nitric oxide (NO), and adenosine (38, 43–45). Adenosine is an important immunosuppressive molecule that is released at high levels by MDSCs in response to hypoxia and inflammation of the tumor microenvironment. In tumors sites, high levels of ectonucleoside triphosphate diphosphohydrolase 1 (CD39) and 5′-nucleotidase ecto (CD73) are expressed by MDSCs, which results in markedly increased levels of adenosine secretion. NK cell antitumor activities are inhibited by adenosine via limiting IFN-γ/TNF-α release, inhibiting Fas ligand and perforin-mediated cytotoxic activity and blocking granzyme exocytosis (38, 46). In patients with hepatocellular carcinoma, a substantial proportion of CD11b^−^CD27^−^NK subsets with an inactive and immature phenotype was reported to accumulate in tumor tissues and render tumor-infiltrating NK cells less tumoricidal, which was also associated with tumor progression (47). High levels of inhibitory molecules, including programmed death ligand 1 (PD-L1) or PD-L2, are expressed on tumor cells, antigen-presenting cells, immunosuppressive cells, and stromal cells in the tumor microenvironment, thus preventing the activation of NK cells through binding with their respective inhibitory receptors on NK cells, which results in exhaustion and dysfunction of NK cells (48, 49). Among the stromal cells, cancer-associated fibroblasts (CAFs) are the major cells that affect the antitumor capacity of NK cells. CAFs secrete IDO or prostaglandin E2 to decrease NK cell expression of NKG2D, and secrete TGF-β to reduce the expression of NKG2D, NKp30, and NKp44 (50, 51). CAFs also inhibit the killing activity of NK cells by downregulating the level of the poliovirus receptor (poliovirus receptor/CD155) (52).

## NK-Cell-Based Tumor Immunotherapy

### Cytokine-Activated NK Cells in Tumor Immunotherapy

NK cells' unique capability to distinguish normal from transformed cells, and their rapid and efficient killing capacity, have led to them becoming an attractive option for tumor immunotherapy and have shown great promise recently. The initial attempts comprised adoptive transfer of peripheral blood-derived autologous or allogeneic NK cells or those stimulated and expanded with cytokines, before injection into patients. In most cases, autologous or allogeneic NK cells were stimulated to activate and proliferate using cytokines (IL-2 or IL-15), in the presence of feeder cells genetically engineered to express costimulatory molecules or cytokines (IL-21 and IL-15) in the culture. The NK cells were then transferred into the patient, followed by the administration of IL-2 to maintain the expansion and function of the infused NK cells (20, 26). Encouraging results were achieved using adoptive transfer of haploidentical NK cells or allogeneic NK cells from killer cell immunoglobulin like receptor (KIR) mismatched donors, leading to graft vs. host disease (GVHD) in patients with hematological cancers. However, these approaches are undesirable to treat patients with solid tumors. The safety of adoptive transfer of autologous or allogeneic NK cells has been proved; however, efficacy is limited by their poor proliferation and persistence *in vivo* and the increase in Tregs related to IL-2 administration (53). Administration of exogenous IL-15 has been shown to improve survival, *in vivo* persistence, and therapeutic efficacy without inducing the survival and expansion of Tregs (54, 55). A human NKG2D–IL-15 fusion protein could efficiently bind to major histocompatibility complex class I polypeptide-related sequence^+^ tumor cells and stimulated NK cell activity by transpresentation of IL-15, thus exhibiting higher control efficiency of the growth of xenografted human gastric cancer via the NK cell recruitment and activation (55). In addition, membrane-bound IL-15 expressed on NK cells (mbIL-15 NK) or *IL15* gene-modified NK cells have been investigated and displayed the ability to survive and proliferate without exogenous cytokines, as well as superior cytotoxicity against both hematological neoplasms and solid tumors (56–59). Moreover, an IL15-IL15Rα-Sushi-Fc fusion protein (ALT-803), termed a superagonist, in which the IL-15Ra sushi domain is complexed with IL-15, potently enhances NK cell survival and cytotoxic activity compared with that induced by native IL-15. In preclinical studies, ALT-803 enhanced memory CD8^+^ T cell subpopulations and specific NK expansion, promoted the secretion of IFN-γ, and improved NK cell function in multiple animal models, including B cell lymphoma, glioblastoma, colon cancer, and ovarian cancer (60–62). Phase I or II clinical trial evaluating its safety and efficacy in patients with both hematological neoplasms (e.g., relapsed or refractory multiple myeloma, acute myelogenous leukemia, acute lymphoblastic leukemia, and myelodysplastic syndromes) and solid tumors (NCT01885897, NCT01946789, NCT02099539, NCT03054909) (63, 64) or in combination with NK cell adoptive therapy (NCT02465957, NCT02890758), or with nivolumab (NCT02523469) (65) are currently ongoing. New expansion methods are being exploited to obtain large numbers of NK cells with enhanced cytotoxic activity (66). Notably, pre-activation of human peripheral blood-derived NK cells (PB-NK) using cytokine combinations, such as IL-18, IL-15, and IL-13, were observed to induce human NK cell differentiation into memory-like NK cells (67–69). These human memory-like NK cells expressed NKG2C, NKG2A, and killer cell lectin like receptor D1 (CD94); however, they had reduced expression of inhibitory KIRs. The pre-activated NK cells express the high-affinity IL-2 receptor and thus are more sensitive to low concentrations of IL-2, such that in IL-2 culture, they proliferate more rapidly and NK cell recovery is higher. The peculiar features of cytokine-induced memory-like NK cells are antigen unspecific with increased proliferative capacity, long-term survival, and *in vivo* persistence, enhanced production of IFN-γ and higher cytotoxicity during *ex vivo* restimulation. Thus, cytokine-induced memory-like NK cells are becoming an attractive tool for antitumor immunotherapy. Recently, several phase I and II clinical trials of cytokine-induced memory-like NK cells have been performed in patients with relapsed or refractory acute myeloid leukemia (AML) after allogeneic hematopoietic cell transplant (NCT03068819, NCT02782546) or myelodysplastic syndrome (NCT01898793) sponsored by Washington University School of Medicine and have shown robust antitumor capacity (67). In addition to PB-NK, human embryonic stem cells, induced pluripotent stem cells (iPSCs), and bone marrow or umbilical-cord blood are being studied as alternative sources of therapeutic NK cells and are showing great promise (70).

### CAR-Modified NK Cells in Tumor Immunotherapy

Recently, the development of chimeric antigen receptor (CAR)-T cells represents a breakthrough in cancer immunotherapy, particularly to treat hematological malignancies. Transfusion of CAR-T cells targeting CD19 to treat relapsed B-cell acute lymphoblastic leukemia ALL (B-ALL) and certain types of relapsed non-Hodgkin's lymphoma obtain as high as 70–90% clinical complete response rates. Thus, CD19-CAR-T cells have been approved by the Food and Drug Administration (FDA) to treat refractory ALL and diffuse large B-cell lymphoma. Currently, multiple CAR-T cells targeting different surface tumor antigens are undergoing clinical development to treat other hematological malignancies and several solid tumors (71, 72). However, there are still a number of obstacles that limit their clinical application. The main problems with CAR-T cell therapy are cytokine release syndrome, neurotoxicity, and on-target/off-tumor effects. To prevent GVHD, CAR-T cells are usually prepared from autologous peripheral blood, which is costly, time consuming, and personalized. NK cells modified with CAR (CAR-NK) are potentially safer than CAR-T cells in that infusion of CAR-NK cells seldom results in cytokine release syndrome because the NK cells secrete mainly restricted levels of granulocyte-macrophage colony-stimulating factor and IFN-γ, while the pro-inflammatory cytokines IL-1 and IL-6 are seldom secreted. The low likelihood of triggering GVHD upon allogeneic infusion means that CAR-NK cells may be prepared as an “off-the-shelf” product and are not restricted to autologous cells. Moreover, natural recognition receptors that recognize stress-induced ligands independent of CARs are retained on CAR-NK cells, including CD226, NKp30, NKp46, NKG2D, and NKp44. NK cells express FcγRIII (CD16) that can mediate ADCC. Therefore, CAR-NK cells are able to induce tumor cell lysis in CAR-dependent and CAR-independent manners, not only further promoting their killing activity but also decreasing the possibility of loss of CAR-targeting antigen-related relapse (73, 74). Intriguingly, activating signaling mediated by CAR is able to overcome the uneducated NK cell hyporesponsiveness, thereby mediating target cell killing. Indeed, CAR-mediated activation is able to overcome the dominant inhibition associated with NKG2A-mediated signaling, thereby enhancing NK cells' antitumor responses (75, 76).

To date, CAR-NK cells are being studied in ongoing clinical trials to evaluate their safety and efficacy for both hematological malignancies and solid tumors, which have shown promising results (Table 1). The main targets for hematological malignancy include CD19, CD22, CD33, and CD7 for relapsed and refractory leukemia and lymphoma (NCT01974479, NCT02742727, NCT02892695, NCT02944162, NCT03056339, NCT03579927, NCT03690310, NCT03692767, and NCT03824951) and B-cell maturation antigen for multiple myeloma (NCT03940833). The targets of current ongoing CAR-NK cells for solid cancer in clinical trials include mucin 1, human epidermal growth factor receptor 2, NKG2D ligands, roundabout guidance receptor 1 (ROBO1), and Mesothelin (NCT02839954, NCT03383978, NCT03415100, NCT03692637, NCT03692663, NCT03931720, NCT03940820, NCT03941457).

**Table 1:** Current clinical trials of CAR-NK cells.

**ClinicalTrials.gov Identifier**	**Malignancy**	**Phase**	**NK source**	**Target**	**Signaling domain of CAR**
NCT02944162	CD33^+^ acute myeloid leukemia	I/II	NK-92 cell	CD33	CD28-4-1 BB-CD3ζ
NCT01974479	B-cell acute lymphoblastic leukemia	II	Primary NK cells	CD19	CD8αTM-4-1 BB-CD3ζ
NCT02742727	Leukemia and Lymphoma	I/II	NK-92 cell	CD7	CD28-4-1 BB-CD3ζ
NCT02892695	Leukemia and Lymphoma	I/II	NK-92 cell	CD19	CD28-4-1 BB-CD3ζ
NCT03056339	B lymphoid malignancies	I/II	Cord blood NK cells	CD19	CD28-CD3ζ
NCT03579927	B lymphoid malignancies	I/II	Cord blood NK cells	CD19	CD28-CD3ζ
NCT01974479	B-cell acute lymphoblastic leukemia	I	Primary NK cells	CD19	4-1 BB-CD3ζ
NCT03940833	Multiple myeloma	I/II	NK-92 cell	BCMA	CD8αTM-4-1 BB-CD3ζ
NCT03692767	Refractory B-cell lymphoma	I	Unknown	CD22	CD244
NCT03690310	Refractory B-cell lymphoma	I	NK-92 cell	CD19	CD244
NCT03824951	Refractory B-cell lymphoma	I	iPS derived NK cells	CD19	CD244
NCT03824964	Refractory B-cell lymphoma	I	Unknown	CD19/CD22	CD244
NCT02839954	MUC1 positive relapsed or refractory solid tumor	I/II	NK-92 cell	MUC1	CD28-4-1 BB-CD3ζ
NCT03383978	Glioblastoma	I	NK-92 cell	HER2	CD28-CD3ζ
NCT03415100	Solid tumors	I	Primary NK cells	NKG2D-Ligand	Unknown
NCT03940820	Solid tumors	I/II	NK-92 cell	ROBO1	CD8αTM-4-1 BB-CD3ζ
NCT03931720	Malignant tumor	I/II	NK-92 cell	ROBO1	CD8αTM-4-1 BB-CD3ζ
NCT03941457	Pancreatic cancer	I/II	NK-92 cell	ROBO1	CD8αTM-4-1 BB-CD3ζ
NCT03692663	Castration-resistant prostate cancer	I	NK-92 cell	PSMA	CD244
NCT03692637	Epithelial ovarian cancer	I	Primary NK cells	Mesothelin	CD244

The main challenges to the clinical applications of CAR-NK cells include their low transfection efficiency, low *in vivo* persistence, difficulty to infiltrate into the solid tumor sites, and suppression from the tumor microenvironment. Although the viral transduction technology for NK cells has been improved recently, the transfection efficiency for primary NK cells remains unsatisfactory (77, 78). Non-viral vectors, an alternative gene transfer systems, particularly the *Sleeping Beauty* (SB) transposon system, could mediate stable transgene expression (79). CAR transgenes have been successfully transfected into T cells via electroporation using the SB transposon system (80, 81). Several phase I clinical trials have been sponsored by MD Anderson Cancer for the treatment of patients with advanced non-Hodgkin's lymphoma, ALL, and chronic lymphocytic leukemia by infusion of SB-modified CD19-specific CAR-T cells following hematopoietic stem cell transplantation (HSCT) (NCT00968760, NCT01362452, NCT01497184, NCT01653717, and NCT02529813). The results identified 84% CAR expression as well as the feasibility and safety of the SB system (81). An UltraCAR-T co-expressing CAR, mbIL15, and a kill switch introduced by SB transposon system using electroporation was approved by FDA for investigational new drug (IND) application to initiate a phase I study for the treatment of patients with advanced platinum-resistant ovarian cancer. The piggyBac transposon system has also been utilized recently to introduce the *CAR* gene into T or NK cells. Nanoparticle delivery technology has been harnessed to improve the transduction efficiency and augment the antitumor efficacy of immunotherapy (82). Using a T-cell-targeted nanoparticle to deliver the *CAR* gene, Smith et al. report one potential novel and interesting technology to establish *in situ* programming of CD19-CAR-T cells to treat leukemia (83). This approach contains several features: anti-CD3e F(ab′)2 fragments targeting T cells were coupled on the surface of nanoparticles; efficient nuclear delivery was achieved by biodegradable polymer functionalized with nuclear localization signals and microtubule-associated sequences; and stable chromosomal integration was facilitated by the piggyBac transposon system. The authors demonstrated that the nanoparticle-reprogrammed CAR-T cells have similar antitumor therapeutic efficacy to conventional lentiviral-based CAR-T therapy in a B-cell ALL mouse model. Moreover, the particle synthesis is relatively simple, the product is stable, and can be lyophilized for extended storage, with low cost (84, 85). The piggyBac transposon system was also used to introduce NKG2D. CAR-presenting vectors were transfected into NK92 cells to generate NKG2D CAR-NK cells, and efficacy was evaluated in a human lung cancer xenograft model. These non-viral engineered NKG2D CAR-NK cells in combination with CD73 blockade showed significant synergistic therapeutic efficacy (86). The SB transposon system to generate CAR-T cells was found to be safe in recent clinical trials (NCT00968760, NCT01362452, NCT01497184, NCT01653717, and NCT02529813). Therefore, it might be feasible to use the SB or piggyBac transposon system combined with nanoparticle delivery technology to establish CAR-NK cells for clinical therapy, although the potential risks and benefits need to be carefully evaluated.

The CAR constructs in most CAR-NK are similar to those in CAR-T cells, except for the unique signaling, and activation features of NK cells. Thus, designing optimized CAR structures suitable for NK cells has been pursued. The NK-cell-activating receptor NKG2D recognizes multiple ligands, such as UL-16-binding proteins, major histocompatibility complex class I chain-related A, and MICB, which are present in increased abundance on the surface of viral-infected cells and many tumor cells. The first NK-cell-based CAR were NKG2D-expressing CAR-NK cells containing a NKG2D–DNAX activation protein 10 (DAP10)–CD3ζ construct, which have shown encouraging results *in vitro* and in a mouse model of osteosarcoma (87). This NKG2D CAR can recognize ~90% of human tumor types that express NKG2D ligands. Notably, several studies have demonstrated that NKG2D ligands are expressed on immunosuppressive cells, such as MDSCs and Tregs; therefore, NKG2D-expressing CAR-NK cells not only directly kill NKG2D^+^ tumor cells but also could eradicate immunosuppressive cells in the tumor environment (88). Other NK-activating receptors (NKp30 and DNAM-1)-based CAR could be designed using a similar strategy, having advantage that one CAR could target several of tumor types that express the corresponding ligands. Prostate stem cell antigen (PSCA)-DAP12 CAR, targeting the prostate stem cell antigen, was expressed in primary NK cells and the YTS-NK cell line and improved the specific cytotoxicity compared with PSCA-CD3ζ-based CAR NK cells (89). Interestingly, no additional costimulatory signaling molecules for *in vitro* cytotoxicity and activation were required by PSCA-DAP12 CAR. A panel of NK-cell-specific CAR constructs comprising various combinations of NK-cell-specific activating domains were screened for NK cell activation recently (90). The results showed that for optimal antigen-specific NK cell signaling and NK-cell-mediated cytotoxicity, the intracellular 2B4 domain, the CD3ζ signaling domain, and the transmembrane NKG2D are necessary. Based on these CAR constructs, mesothelin-targeted CAR-NK cells were constructed using human-induced pluripotent stem cells, and the antitumor activity was examined *in vitro* and using a murine ovarian cancer xenograft model. The NK-specific CAR-NK cells exhibited a markedly augmented cytolytic capacity, a significantly lower tumor burden, and prolonged survival compared with a traditional T-cell CAR construct expressing CAR-NK cells (90). Notably, the *in vivo* expansion and survival of the NK cells was improved as a result of the NK-cell-specific CAR-mediated signaling.

To increase the specificity, efficacy, and safety of CAR-NK cells, as well as to overcome suppression by the tumor microenvironment, the strategies and experience from CAR-T studies could be applied to CAR-NK research. The targets used for CAR-NK research in preclinical studies include CD19, CD20, CD138, CD5, CD2 subset 1 (CS1), NKG2D ligand, glucosylceramidase beta (GD2), HER-2, epidermal growth factor receptor (EGFR), EGFRvIII, epithelial cell adhesion molecule 1, glypican 3, and guanine nucleotide-binding protein alpha-7 (91). For example, IL15 was introduced into CAR design to increase CAR-NK cells' antitumor capacity and persistence (92). CAR-NK cells can be engineered with chemokine receptors or chemokines to enhance NK cell homing and infiltrating to tumor sites.

The sources of NK cells include PB-NK cells, umbilical cord blood NK cells, and the NK cell line NK-92. However, the use of PB-NK cells is limited because primary NK cells are generally hard to genetically modify; therefore, the transduction efficacy is very low, even with viral vectors, and in the adoptive transfer setting, the allogeneic NK cells can survive for only a few weeks, limiting their antitumor efficacy (93). Recently, stem-cell-derived NK cells, such as those induced from human embryonic stem cells or iPSCs, have showed promise as candidates to develop off-the-shelf adoptive NK cell therapy. They have a similar phenotype and function to primary NK cells with an efficient capacity to kill both hematological malignancies and solid tumor cells. More importantly, they are homogenous, reproducible, and amenable to genetic modification, and can be easily expanded on the clinical scale (70, 94–96).

Notably, mesothelin-targeted CAR-NK cells prepared from human iPSCs showed significantly inhibited tumor growth, prolonged survival, and markedly augmented cytotoxicity in a xenograft model of ovarian cancer (90). Thus, iPSC-derived CAR-NK cells could be a promising resource to generate standardized, off-the-shelf, and clinical-scale adoptive immunotherapeutic products. Moreover, human iPSC-NK cells could be genetically modified to further improve their *in vivo* persistence, overcome the immunosuppressive tumor microenvironment, and enhance their antitumor capacity. iPSC-NK cells could also be harnessed in combination with other therapies (such as checkpoint blockade). Clinical trials treating cancer with human iPSC-NK cells are currently under IND application. The safety concerns for iPSC-NK cells include possible immune responses against allogenic iPSCs and their potential for malignant transformation. HLA-E-expressing or HLA knockout together with overexpression of CD47 hypoimmunogenic iPSCs have been generated recently to evade immune rejection by host cells (97, 98).

### Antibody-Based NK Cell Therapy

#### ADCC of NK Cells Mediated by Tumor-Specific Antibodies

One of the main killing mechanism for antitumor effects of NK cells is ADCC, which involves the binding of the Fc portion of an antibody (Ab) to CD16 on NK cells. Most tumor-targeting Ab drugs exert their antitumor effect by promoting NK-cell ADCC. Studies have tried to increase the affinity of Abs to Fc receptors on NK cells to enhance ADCC by modifying Abs via mutagenesis or glycosylation (99). Expression high-affinity CD16 FcγRIII in NK cells is also a strategy used to enhance ADCC-mediated antitumor effects. To enhance NK-mediated ADCC using Cetuximab, Trastuzumab, and Pertuzumab, IL-2 and the high-affinity CD16 FcγRIIIa (158V) allele were engineered into NK-92 cells (named haNK) (100). Phase I and II clinical trials of haNK, alone, or in combination with anti-PD-L1 Ab (avelumab), a cancer vaccine, or super-IL-15, are ongoing for the treatment of triple negative breast cancer, squamous cell carcinoma, Merkel cell carcinoma, pancreatic cancer, and other types of cancers (NCT03027128, NCT03387085, NCT03387111, NCT03853317, and NCT03586869). CAR-modified haNK (also named target-activated NK-92) cells targeting CD19 and PD-L1 are currently under investigation and have recently been approved for IND by the FDA.

#### Potentiation of NK Cell Activity Using Bi- or Trispecific Killer Engagers

Molecules comprising single-chain variable fragments against activating receptors on NK cells and tumor-associated antigen, termed bi- or trispecific killer cell engagers (BiKEs or TriKEs), have been developed to create an immune connection between NK and tumor cells, thus promoting NK-mediated killing of tumor cells. Several NK-based BiKEs or TriKEs are currently in preclinical and clinical development. CD16-directed BiKEs CD16 × 19 and CD16 × 33 and TriKE CD16 × 19 × 22 were shown to specifically stimulate NK cell activation via CD16, which triggers NK cell cytolytic activity and cytokine secretion to fight lymphoma and leukemia (101, 102). A combination comprising an ADAM metallopeptidase domain 17 inhibitor further enhanced the function of NK cells by preventing CD16 shedding from the membrane of NK cells (102). Multiple other CD16-directed BiKEs, such as CD16 × Erb-B2 receptor tyrosine kinase 2, CD16 × human epidermal growth factor receptor 2/neu, CD16 × EGFR, CD16 × carcinoembryonic antigen, and CD16 × EpCAM, are under investigation (103). A bi-specific Ab-recognizing CS1-NKG2D, which comprises an anti-NKG2D scFv and an anti-CS1 scFv, showed enhanced cytotoxicity and cytokine production from NK cells and significantly prolonged their survival in a multiple myeloma xenograft NOD-SCID^IL2γ*c*−/−^ (NSG) mouse model (104). A novel CD30/CD16A tetravalent bispecific Ab (AFM13), which has a longer half-life compared with that of other bi-/trispecific Abs, is currently under evaluation in a phase II clinical trial to treat patients with relapsed/refractory Hodgkin's lymphomas (NCT03192202) (105, 106).

To augment the *in vivo* expansion and survival of NK cells, a TriKE was constructed having a modified IL-15 cross-linker (107, 108). CD16 × IL-15 × CD33 displayed markedly enhanced NK cytotoxicity against AML and better cell persistence *in vivo* compared with those of BiKEs. Importantly, it is expected to target both malignant cells and CD33^+^ MDSCs, thus exerting more efficient antitumor effect (107). Currently, CD16 × IL-15 × CD33 TriKE is being evaluated in phase I and II clinical trials in patients with advanced systemic mastocytosis, refractory/relapsed acute myeloid leukemia, or CD33-expressing high-risk myelodysplastic syndromes (NCT03214666). CD16 × IL-15 × Epcam TriKE, CD16 × IL-15 × CD133 TriKEs, and CD16 × IL-15 × CD133 × Epcam TetraKE are under investigation to treat colorectal cancer and have shown significantly enhanced cytokine secretion, NK cell proliferation, and lytic degranulation (108–110). More recently, trifunctional NK cell engagers targeting two activating receptors, NKp46 and CD16, on NK cells and a tumor antigen (CD19, CD20, or EGFR) on cancer cells were generated by Prof. Vivier and his colleagues (111). By cotargeting NKp46 and CD16, which led to full NK cell activation, the trifunctional NK cell engagers showed more efficient promotion of NK cell activation and cytotoxicity *in vitro* and more effective control of tumor growth in mouse models of solid and invasive tumors compared with those of current clinically available therapeutic antibodies. Remarkably, TriKEs represent a cost-effective and versatile platform for the incorporation of novel targeting molecules and could potentially boost NK cell function. For example, introducing Abs against checkpoint receptors or TGF-β to construct TriKEs might enhance NK cell efficacy by reversing the immunosuppression from the tumor microenvironment (TME) (112).

#### Releasing the Inhibition on NK Cells by Targeting the Immune Checkpoints

Recently, monoclonal antibodies (mAbs) targeting immune checkpoint ligands or receptors have been applied to markedly enhance the tumor-suppressed T-cell immune response, leading to the improved control of several types of cancer. Similarly, a variety of immune checkpoints receptors are expressed in NK cells, including KIRs, NKG2A, T-cell immunoglobulin- and mucin-domain-containing molecule 3, programmed cell death 1 (PD-1), lymphocyte activation gene-3, and T-cell immunoreceptor with immunoglobulin and immunoreceptor tyrosine-based inhibition motif domains (TIGIT). These inhibitory receptors are often induced or upregulated on tumor-infiltrating NK cells and affect the antitumor function of NK cells upon interaction with their respective ligands, even leading to dysfunction or exhaustion of NK cells (113). Functionally exhausted NK cells show less proliferation, reduced cytolytic activity, and downregulated cytokine secretion, thus losing the ability to attack tumor cells. Therapeutic blockade of the immune checkpoint receptors or ligands with mAbs can restore the antitumor function of NK cells, which have shown great promise for NK-cell-based tumor immunotherapy (114).

The first attempt was blockade of HLA-KIR interactions to ameliorate NK cell inhibition and enhance NK cell activity using the anti-KIR antibody, IPH2101, to treat leukemia, lymphoma, and multiple myeloma, which demonstrated efficacy and safety (115). The second generation, fully human immunoglobulin G4 anti-KIR antibody, IPH2102, was well-tolerated in patients with hematological malignancies and solid tumors (116). Anti-KIR antibodies combined with other immunotherapies, for example, anti-PD-1 and anticytotoxic T-lymphocyte-associated protein 4 Abs, to treat solid tumors are currently ongoing (NCT03203876, NCT03347123).

In various cancers, such as cervical cancer, breast cancer, hepatocellular carcinoma, and lung cancer, high NKG2A expression was identified inn tumor-infiltrating NK and T cells, which contributes to exhaustion of these cells and predicts a poor prognosis (49). Therapeutic blockade of NKG2A could improve NK cell dysfunction in both hematological malignancies and solid tumors (117, 118). In particular, a humanized anti-NKG2A Ab, Monalizumab (IPH2201), was reported to augment NK cell activity against certain types of tumor cell (B-cell lymphoma, solid tumors, and T-cell lymphoma) and rescue the effector and activation functions CD8^+^ T and NK cells, especially in combination with PD-L1 blockade (118). A phase I and II clinical trial of IPH2201 and Cetuximab, with or without anti-PD-L1 Abs, in patients with human papillomavirus (+) and (–) recurrent or metastatic squamous cell carcinoma of the head and neck is ongoing (NCT02643550). Its safety was evaluated as well-tolerated for the combination therapy. The interim results of the phase II trial for treatment efficacy showed that combination therapy comprising IPH2201 and Cetuximab resulted in 31% partial response, 54% stable disease, and 11% progressive disease (118). Therefore, anti-NKG2A mAbs are proposed as promising checkpoint inhibitors that could enhance antitumor immunity via unleashing the potential of both T and NK cells (113, 119).

Tumor-infiltrating NK cells from patients with various cancers induce PD-1, which causes inducing functional failure of activated NK cells, which is associated with poor prognosis (120, 121). Blocking PD-1/PD-L1 signaling markedly increased the cytotoxicity and cytokine production of NK cells and significantly suppressed tumor growth *in vivo* (121). Several antibodies targeting PD-1 and PD-L1 have been used in clinical practice, and several others are under investigation to treat different solid and hematological malignancies (122). Various combination therapies of anti-PD-1/PD-L1 Abs with other checkpoint blockades, antiangiogenic bevacizumab, or chemotherapy have been explored recently in clinical trials. A current phase II clinical trial is assessing the effect of Pembrolizumab (an anti-PD-1 Ab) to induce changes to NK cell exhaustion and function in patients with unresectable melanoma at stage III or stage IV (NCT03241927).

Inhibitory receptors TIGIT and CD96 are regarded as new checkpoint receptors for NK cells and T cells. TIGIT competes with CD226 (DNAM-1) (an NK activating receptor) for the same set of ligands CD112 [Poliovirus receptor-related 2 (Herpesvirus entry mediator B)] and CD155 (poliovirus receptor), while CD96 shares binding of CD155 with CD226 and TIGIT but also binds to CD111 to directly inhibit NK cell function. Similar to the CD28/cytotoxic T-lymphocyte-associated protein 4 pathway, TIGIT and CD96, together with CD226, form a pathway in which CD226 functions as a costimulatory receptor, whereas TIGIT and CD96 act as coinhibitory receptors (123). The balance among the three receptors fine tunes the immune response against tumors. In patients with cancer, TIGIT and CD96 are upregulated in tumor-associated NK cells and promote NK cell functional exhaustion, accompanied by poor cytolytic potential and impaired cytokine production (124, 125). Blockade of TIGIT with Abs could effectively reverse NK cell exhaustion and enhance NK-cell-dependent antitumor immune responses in several tumor-bearing mouse models. Intriguingly, blockade of TIGIT could further promote tumor-specific T-cell immune responses and improve memory responses to tumor rechallenge (11). Blocking CD96–CD155 interaction could reverse NK- and T-cell exhaustion, and restore both NK- and T-cell-mediated antitumor immunity (125, 126). Recently, IL-1 receptor 8 long isoform (also known as single immunoglobulin IL-1R-related receptor) was identified as an NK cell checkpoint protein that regulates NK cell maturation and antitumor activity. Genetic blockade of IL-1 receptor 8 long isoform induces NK-cell-mediated resistance in hepatic carcinogenesis and liver or lung metastasis (127). Currently, the safety and efficacy of anti-TIGIT Ab, alone or in combination with anti-PD-1 or anti-PD-L1 Abs, are being evaluated in phase I and II clinical trials in patients with locally advanced or metastatic solid tumors, e.g., renal cell carcinoma, non-small cell lung cancer, breast cancer, squamous cell carcinoma of the head and neck, melanoma, and colorectal cancer (NCT03119428, NCT03563716, NCT03628677). Blocking Abs for other checkpoint proteins, including T-cell immunoglobulin- and mucin-domain-containing molecule 3 and lymphocyte activation gene-3, alone or in combination with other therapeutic approaches, are also currently in progress in patients in clinical trials or are under investigation for therapy of both hematological neoplasms and various solid tumors (e.g., NCT02060188, NCT02817633, NCT03307785, NCT03680508, NCT03250832, NCT03489369, NCT03625323, NCT03662659). Current clinical trials of checkpoint blockade antibodies to enhance NK cell antitumor efficacy are summarized in Table 2.

**Table 2:** Current clinical trials of checkpoint blockade molecules.

**Checkpoint Receptor**	**Ab**	**Combination**	**Malignancy**	**Phase**	**ClinicalTrials.gov Identifier**
KIR	IPH4102		Cutaneous T-cell lymphoma	I	NCT02593045
	Lirilumab	Nivolumab (anti-PD1 Ab)	Bladder cancer	I	NCT03532451
	Lirilumab	Nivolumab (anti-PD1 Ab) Ipilimumab (anti-CTLA-4 Ab)	Advanced and/or metastatic solid tumors	I	NCT03203876
	Lirilumab		Advanced solid tumors	I/II	NCT01714739
	Lirilumab	Nivolumab (anti-PD1 Ab) Ipilimumab (anti-CTLA-4 Ab)	Advanced (metastatic and/or unresectable) Refractory Solid Tumors	I/II	NCT01714739
	Lirilumab	Nivolumab (anti-PD1 Ab) Ipilimumab (anti-CTLA-4 Ab)	Solid tumors	I/II	NCT03347123
	Lirilumab	Nivolumab (anti-PD1 Ab) Ipilimumab (anti-CTLA-4 Ab) Daratumumab (anti-CD38 Ab)	Multiple myeloma	I/II	NCT01592370
	Lirilumab	Nivolumab (anti-PD1 Ab)	Squamous cell carcinoma of the head and neck	II	NCT03341936
	Lirilumab	Rituximab (anti-CD20 Ab)	Lymphocytic leukemia	II	NCT02481297
NKG2A	Monalizumab		Hematologic malignancies	I	NCT02921685
	Monalizumab		Gynecologic cancer	I	NCT02459301
	Monalizumab		Chronic lymphocytic leukemia	I/II	NCT02557516
	Monalizumab	Cetuximab (anti-EGFR Ab)	Head and neck neoplasms	I/II	NCT02643550
	Monalizumab	Durvalumab (anti-PD-L1 Ab)	Advanced solid tumors	I/II	NCT02671435
	Monalizumab	Durvalumab (anti-PD-L1 Ab) Oleclumab (anti-CD73 Ab)	Non-small cell lung cancer	II	NCT03833440
	Monalizumab	Durvalumab (anti-PD-L1 Ab) Oleclumab (anti-CD73 Ab)	Stage III non-small cell lung cancer	II	NCT03822351
TIGIT	OMP-313M32		Locally advanced cancer, metastatic cancer	I	NCT03119428
	AB154	AB122 (anti-PD1 Ab)	Advanced malignancies	I	NCT03628677
	MTIG7192A	Atezolizumab (anti-PD1 Ab)	Non-small cell lung cancer	II	NCT03563716
TIM-3	Sym023		Metastatic cancer, solid tumor, lymphoma	I	NCT03489343
	TSR-022		Advanced or metastatic solid tumors	I	NCT02817633
	RO7121661		Solid tumors, metastatic melanoma, non-small cell lung cancer	I	NCT03708328
	LY3321367		Solid tumor	I	NCT03099109
	MBG453	Spartalizumab (anti-PD1 Ab)	Glioblastoma multiforme	I	NCT03961971
	MBG453		Acute myeloid leukemia	I	NCT03940352
	MBG453	PDR001 (anti-PD1 Ab)	Leukemia	I	NCT03066648
	INCAGN02390		Advanced malignancies	I	NCT03652077
	BGB-A425	Tislelizumab (anti-PD1 Ab)	Locally advanced or metastatic solid tumors	I/II	NCT03744468
	MBG453	PDR001 (anti-PD1 Ab)	Advanced malignancies	I/II	NCT02608268
	TSR-022	TSR-042 (anti-PD1 Ab)	Liver cancer	II	NCT03680508
PD-1	Nivolumab Pembrolizumab Atezolizumab	IPSC-derived NK Cells	Advanced solid tumors	I	NCT03841110
	Pembrolizumab	DC-NK Cells	Solid tumor	I	NCT03815084
	Nivolumab	NK Cells	Renal cell carcinoma	I	NCT03891485
	Pembrolizumab	Allogeneic NK Cells	Biliary tract cancer	I/II	NCT03937895
	Pembrolizumab		Lymphoma	I/II	NCT02535247
	PD-1 Ab	NK Cells	Non-small cell lung cancer	II	NCT03958097
	Avelumab	haNK™	Merkel cell carcinoma	II	NCT03853317
LAG-3	Sym022		Metastatic cancer, solid tumor, lymphoma	I	NCT03489369
	TSR-033		Advanced solid tumors	I	NCT03250832
	BMS-986016	Nivolumab (anti-PD1 Ab)	Glioblastoma	I	NCT02658981
	REGN3767	REGN2810 (anti-PD1 Ab)	Malignancies	I	NCT03005782
	MGD013		Advanced solid tumors, hematologic neoplasms	I	NCT03219268
	IMP321		Solid tumors	I	NCT03252938
	Relatlimab	Nivolumab (anti-PD1 Ab)	Advanced solid tumors	I	NCT02966548
	Relatlimab	Nivolumab (anti-PD1 Ab)	Gastric cancer, cancer of the stomach	I	NCT03662659
	FS118		Advanced cancer, metastatic cancer	I	NCT03440437
	BMS-986016		Hematologic neoplasms	I/II	NCT02061761
	Relatlimab		Solid tumors	I/II	NCT01968109
	IMP321	Pembrolizumab (anti-PD1 Ab)	Non-small cell lung carcinoma, head and heck carcinoma	II	NCT03625323
	Relatlimab	Nivolumab (anti-PD1 Ab)	Melanoma	II	NCT03743766
	Relatlimab	Nivolumab (anti-PD1 Ab)	Advanced chordoma	II	NCT03623854

#### Other Strategies to Overcome the Suppression by the Tumor Microenvironment

The TME is a major obstacle for ensuring the optimum antitumor activity of NK cells, in which immunosuppressive cells and molecules limit NK cell activity. In addition, to overcome the suppression from inhibitory receptors or ligands by checkpoint blockade, neutralizing, or blocking suppressive cytokines secreted by tumor cells, immunosuppressive cells, or stromal cells in the TME is another key strategy. One of the most important immunosuppressive cytokine is TGF-β, which is secreted by tumor cells, Tregs, MDSCs, and other stromal cells in the TME to hamper the antitumor immune response. NK cell antitumor function is inhibited by TGF-β-mediated downregulation of the expression of NK-activating receptors NKp30 and NKG2D and also reduces the expression of NKG2D ligands on tumor cells, thus suppressing NK-mediated cytotoxic capacity and IFN-γ secretion (103). TGF-β also affects the development and differentiation of human NK cell subsets (128). Blockade of TGF-β signaling or neutralization of TGF-β could prevent NKG2D downregulation and restore the antitumor function of NK cells. Therapies that interrupt TGF-β signaling to enhance NK cell antitumor capacity are currently in clinical trials or are under investigation. The safety and efficacy of human TGF-β neutralizing mAb Fresolimumab (GC1008) and TGFβR1 inhibitor Galunisertib (LY2157299) have been evaluated in a phase I clinical trial to treat patients with advanced malignant melanoma, renal cell carcinoma, and other advanced solid tumors, and have obtained acceptable tolerability and safety results (NCT00356460, NCT01722825). Importantly, Galunisertib therapy could restore NKG2D and NKp30 expression on activated NK cells and enhanced NK cell cytotoxicity. Knockdown of *TGFBR2* or *SMAD3* in NK cells, engineering CB NK cells to express TGF-β dominant negative receptor II, or modifying NK cells using CAR containing TGF-β type II receptor extracellular and transmembrane domains, and the intracellular domain of NKG2D, are all under investigation and have shown great promise for the recovery of tumor-suppressed NK cell antitumor activity to treat patients with solid tumors (129–131).

## Conclusion and Perspectives

Although there are still some challenges that limit the widespread use of NK-cell-based therapies, particular for solid tumors, advances in *ex vivo* expansion and activation technologies, genetic modification, and nanoparticle delivery technology will lead to novel therapeutic strategies to overcome the immune suppression from the TME of solid tumors, indicating that that NK cell therapy is achievable and promises to become a powerful method to treat cancers. In certain respects, NK cells have unique advantages over conventional T cells (73, 132). Notably, the use of NK cells could result in off-the-shelf allogeneic products to treat patients, thereby eliminating the necessity for personalized and patient-specific products in current CAR-T cell therapies. NK-cell-based therapy provides an alternative or complementary immunotherapy approach to T-cell therapy. It is worth exploring the utility of the selective expansion of suitable NK cell subsets and their delivery to the corresponding type of tumor. The longer lifespan of memory-like NK cells compared with that of PB-derived NK cells may make them ideal sources for NK cell therapy. Recently, CMV-induced adaptive memory NK cells were demonstrated as relatively resistant to Tregs and MDSCs' suppressive effects, which might have important clinical implications (133, 134). Remarkable benefits might be achieved by engineering the CAR protein into memory-like NK cells or a specific NK cell subset. In fact, CD19-specific CAR-engineered NKG2C^+^CD57^+^ adaptive NK cells showed an enhanced ability to kill CD19^+^ tumor cells compared with that of other NK subsets (75). Different combinations of multiple strategies could play an increasingly prominent role in clinical practice. For example, the use of checkpoint blockade to overcome the immunosuppressive effect within the TME could increase the endogenous NK cell antitumor ability and the function of adoptive transferred NK cells. Therefore, it is reasonable to combine checkpoint inhibition with adoptive transfer of allogeneic NK cells or CAR-transduced NK cells. Checkpoint receptor blockade in conjunction with BiKEs or TriKEs could increase antigen specificity and reverse immunosuppression, thus further enhancing the antitumor responses of NK cells.

## Author Contributions

CZ and YH designed and conceived the review. YH and CS performed the literature search and analysis. CZ wrote the initial draft of the manuscript together with YH and CS. All authors revised and approved the final submitted version of the manuscript.

### Conflict of Interest

The authors declare that the research was conducted in the absence of any commercial or financial relationships that could be construed as a potential conflict of interest.
